# Multi-site observational evaluation of Breast AI™-supported ultrasound risk stratification in breast cancer assessment pathways

**DOI:** 10.1371/journal.pone.0357975

**Published:** 2026-09-15

**Authors:** Kathryn Malherbe, Carol A. Benn, Gerhard Botha

**Affiliations:** 1 Malherbe Imaging Inc., Diagnostic Imaging, Pretoria, South Africa; 2 Department of Immunology, University of Witwatersrand, Johannesburg, South Africa; 3 Department of Family Medicine, University of Pretoria, Pretoria, South Africa; Nelson Mandela African Institute of Science and Technology, UNITED REPUBLIC OF TANZANIA

## Abstract

**Purpose:**

To evaluate the diagnostic performance metrics, referral patterns, and real-world implementation characteristics associated with a Breast AI™-supported ultrasound assessment pathway across multiple heterogeneous healthcare settings.

**Materials and methods:**

This prospective multi-site observational study included 1,129 women undergoing routine or symptom-driven breast assessment across five healthcare facilities in South Africa between April 2023 and April 2025. Breast AI™ was used as an adjunctive ultrasound-based clinical decision-support tool alongside standard clinical assessment and breast ultrasound imaging. Referral outcomes and histopathological diagnoses were recorded where clinically indicated. Diagnostic performance metrics, including sensitivity, specificity, positive predictive value (PPV), and negative predictive value (NPV), were calculated using predefined dichotomised Breast AI™ risk categories. Site-level comparisons were assessed using chi-square analysis.

**Results:**

Among 1,129 clinical assessments, 417 patients underwent referral for further diagnostic evaluation, with 405 histopathologically confirmed malignancies identified. Referral-associated malignancy rates remained relatively consistent across participating healthcare sites, with no statistically significant site-level differences observed (χ² = 1.67, p = 0.795). Diagnostic performance analysis demonstrated a sensitivity of 99.3% (95% CI: 97.8–99.8), specificity of 69.8% (95% CI: 66.3–73.1), PPV of 64.7% (95% CI: 61.2–68.0), and NPV of 99.4% (95% CI: 98.3–99.8). Higher Breast AI™ risk classifications were more frequently associated with invasive ductal carcinoma and invasive lobular carcinoma, whereas approximately 30% of ductal carcinoma in situ cases were classified within the low-risk category. The median interval between Breast AI™ assessment and histopathological confirmation was 18 days (IQR: 10–32 days).

**Conclusion:**

In this prospective observational multi-site cohort, Breast AI™-supported ultrasound assessment demonstrated high observed negative predictive value and reproducible risk stratification across heterogeneous healthcare environments. However, interpretation of diagnostic accuracy metrics is limited by differential histopathological verification and the absence of long-term interval cancer follow-up among low-risk patients. The findings support the feasibility of integrating AI-supported ultrasound assessment into resource-variable clinical settings, while highlighting the need for future comparative, longitudinal, and health-economic evaluation studies

## Introduction

Artificial intelligence (AI) is increasingly being integrated into radiology workflows through the development of scalable diagnostic support systems capable of assisting image interpretation and clinical risk stratification [1,2]. In breast imaging, AI-supported technologies have demonstrated potential utility in improving lesion characterization, supporting early cancer detection, and assisting clinical decision-making processes [3–6]. These technologies are of particular interest in resource-constrained healthcare settings, where shortages of specialist personnel and diagnostic infrastructure may delay diagnosis and treatment initiation.

Breast cancer remains a major global public health challenge and is a leading cause of cancer-related mortality among women worldwide [7,8]. The burden is particularly pronounced in low- and middle-income countries (LMICs), where limited access to screening programs, delayed presentation, restricted imaging availability, and shortages of trained healthcare professionals contribute to poorer clinical outcomes and advanced-stage disease at diagnosis [8–10]. Early detection remains critical for improving survival outcomes and facilitating clinical downstaging; however, conventional mammography-based screening programs are often difficult to implement sustainably in LMIC settings because of infrastructure limitations, cost constraints, and workforce shortages [8,9].

Alternative imaging pathways incorporating clinical breast examination (CBE) and ultrasound have therefore gained increasing relevance within these environments [8,11]. AI-enhanced ultrasound systems may provide an adjunctive approach for supporting risk stratification and referral decision-making, particularly in settings with variable access to specialist breast radiology services. The Breast AI™ system evaluated in this study integrates ultrasound imaging with AI-derived probabilistic risk classification to support clinical assessment workflows. The system analyses ultrasound-derived imaging features associated with malignancy probability and categorises findings into predefined risk groups intended to assist, rather than replace, clinician interpretation and referral decisions [1,7,11,12].

Despite the growing interest in AI-supported diagnostic systems, important implementation challenges remain. These include variability in infrastructure readiness, training requirements, software integration, quality assurance processes, and the operational feasibility of deploying AI-supported imaging tools across heterogeneous healthcare environments. Such considerations are particularly relevant in LMIC settings, where healthcare disparities, infrastructure variability, and workforce limitations may affect the scalability, reproducibility, and long-term sustainability of AI-assisted diagnostic program. Furthermore, while previous studies have demonstrated promising diagnostic performance metrics for AI-assisted breast imaging systems, comparatively limited evidence exists regarding their implementation within prospective multi-site real-world clinical environments [13–16].

The present study was designed as a prospective observational multi-site evaluation of a Breast AI™-supported ultrasound assessment pathway implemented across five diverse healthcare facilities in South Africa. The study included community-based, rural, tertiary, and private-sector clinical settings to evaluate the feasibility of integrating AI-supported ultrasound risk stratification into heterogeneous healthcare environments. This approach is particularly relevant within the South African context, where younger age at presentation, delayed diagnosis, geographic barriers to care, and disparities in access to breast imaging services continue to contribute to poor breast cancer outcomes [9,10,17,18].

Data from the African Breast Cancer–Disparities in Outcomes (ABC-DO) prospective cohort study highlighted the substantial burden of breast cancer among younger women in sub-Saharan Africa, with women younger than 40 years demonstrating particularly poor five-year survival outcomes [17,18]. Contributing factors included advanced-stage presentation, comorbid disease burden, and restricted access to diagnostic and treatment infrastructure. Similar observations have been reported across East African LMIC settings, where limited mammographic screening capacity and shortages of diagnostic services continue to impede early detection efforts [8–10]. Studies from Malawi and other LMIC regions have additionally demonstrated the potential value of clinical breast examination and ultrasound-based assessment pathways for facilitating earlier clinical downstaging in resource-limited populations [8].

Within this context, the current study aimed to evaluate the diagnostic performance and referral characteristics associated with Breast AI™-supported ultrasound assessment within routine clinical practice. Specifically, the objectives of the study were:

1. To evaluate diagnostic performance metrics associated with Breast AI™-supported ultrasound risk stratification across multiple healthcare sites.
2. To assess referral patterns and downstream histopathological outcomes associated with predefined AI-derived risk categories.
3. To explore the feasibility of integrating AI-supported ultrasound assessment within heterogeneous and resource-variable healthcare environments.

## Study design

This study was designed as a prospective observational cohort study conducted across five healthcare sites between 1 April 2023 and 30 April 2025. The Breast AI™ system was deployed as an adjunctive clinical decision-support tool during routine breast ultrasound assessments. Importantly, Breast AI™ outputs did not determine definitive clinical management or override clinician judgement. All referral and management decisions were made by qualified healthcare professionals in accordance with existing site-specific clinical protocols. As such, the study did not introduce an interventional change to standard clinical care pathways and meets the criteria for an observational diagnostic accuracy study.

The specific periods of data collection for each of the five sites are indicated below, the variation in site commencement dates reflected differences in institutional ethics approval timelines, site onboarding processes, and operational availability of participating clinical teams across provinces for each site and availability of data collection site members across the various provinces:

Site 1: 19 July 2024−30 April 2025
Site 2: 1 April 2024–30 April 2025
Site 3: 1 May 2024−30 April 2025
Site 4: 1 July 2024–30 April 2025
Site 5: 1 July 2023–30 October 2024

The study was conducted in accordance with stringent ethical and regulatory standards. Ethical approval was obtained from the relevant institutional research ethics committees prior to study initiation, including the University of Pretoria and University of Cape Town research ethics structures where applicable. All participants provided informed consent before inclusion in the study, and all study procedures adhered to the principles outlined in the Declaration of Helsinki and associated ethical guidelines governing human participant research.

The present investigation was designed as a prospective observational implementation study evaluating Breast AI™-supported ultrasound assessment within routine clinical practice. Because the study did not involve randomisation, therapeutic intervention allocation, or experimental modification of patient management pathways, prospective clinical trial registration was not undertaken. Breast AI™ was utilised as an adjunctive clinical decision-support tool within existing diagnostic workflows, and all clinical management decisions remained under the responsibility of the attending healthcare professionals according to standard institutional protocols.

The study cohort comprised 1,129 female patients undergoing routine or symptom-driven breast assessment across the participating healthcare sites. Participants received standard clinical care appropriate to their respective institutions, including clinical breast examination (CBE), specialist consultation where indicated, and breast ultrasound imaging. Breast AI™-supported ultrasound assessment was performed concurrently as an adjunctive decision-support tool during routine clinical workflows and was not considered part of the established standard-of-care diagnostic pathway at any participating site.

The sample size was determined based on prospective multi-site recruitment feasibility across the study period rather than through formal hypothesis-driven power calculation, as the study was designed as an observational implementation evaluation rather than a comparative interventional trial. Nevertheless, inclusion of more than 1,100 participants allowed estimation of key diagnostic performance metrics, including sensitivity, specificity, positive predictive value (PPV), and negative predictive value (NPV), with acceptable precision and corresponding 95% confidence intervals. The multi-site design additionally enabled assessment of Breast AI™-supported assessment across heterogeneous healthcare settings with variable patient demographics, referral environments, and diagnostic resource availability.

Patients were included if they presented for routine breast cancer clinical assessment or were referred for imaging due to symptoms such as palpable lumps or mastalgia. Patients with a previous history of breast cancer, currently on primary endocrine therapy or incomplete imaging data were excluded from the study. The cohort had a mean age of 52.4 ± 11.8 years (range: 30–80 years) and included both pre- and post-menopausal women. Participants were recruited from five heterogeneous healthcare facilities representing diverse clinical environments, referral pathways, patient demographics, and levels of diagnostic resource availability within South Africa. The inclusion of multiple healthcare settings was intended to evaluate the feasibility of integrating Breast AI™-supported ultrasound assessment across variable real-world clinical environments.

**Site 1,** Daspoort Poli Clinic is a community-based primary healthcare facility serving predominantly lower-income and medically underserved populations with limited access to specialist breast imaging services. The clinic operates within a resource-constrained environment where diagnostic infrastructure and specialist referral capacity are restricted. Patients typically present through primary healthcare referral pathways, often with symptomatic breast complaints requiring initial clinical assessment and triage.

**Site 2,** Quadcare Clinic is a private-sector outpatient diagnostic facility serving predominantly insured and self-funded patient populations within an urban setting. The clinic provides access to structured referral pathways, specialist imaging services, and comparatively greater diagnostic resource availability. Patients commonly present through physician referral or self-initiated screening and diagnostic consultations.

**Site 3,** The Breast Care Centre of Excellence is a high-volume urban multidisciplinary breast imaging and diagnostic centre managing a broad spectrum of symptomatic and screening-related breast pathology. The facility serves a mixed referral population and incorporates specialist breast imaging, surgical consultation, and coordinated oncological referral services. The centre represents a high-throughput clinical environment with established breast diagnostic workflows and advanced imaging infrastructure.

**Site 4,** A District Hospital is a rural healthcare facility serving geographically dispersed and historically underserved populations with variable access to specialist diagnostic services. The hospital manages patients within a resource-variable public-sector environment where referral distances, healthcare access limitations, and delayed clinical presentation may affect diagnostic pathways. Breast imaging services are integrated into broader district-level healthcare delivery systems.

**Site 5,** Groote Schuur Hospital is a tertiary academic referral centre with advanced diagnostic, surgical, and oncological capabilities. The institution manages complex breast pathology referrals from secondary and regional healthcare facilities and serves a large and demographically diverse patient population. The hospital incorporates specialist multidisciplinary breast assessment pathways, histopathological services, and tertiary-level imaging infrastructure within a high-acuity academic healthcare environment.

Statistical analysis was performed using SPSS, Statistical Package for the Social Sciences(version 28.0; IBM Corp., Armonk, NY, USA) and Python-based validation scripts. Descriptive statistics were used to summarize patient demographics, referral outcomes, and histopathological diagnoses.

Diagnostic accuracy metrics for Breast AI™ were calculated using standard definitions, including sensitivity, specificity, positive predictive value (PPV), and negative predictive value (NPV). For the purposes of diagnostic accuracy analysis, Breast AI™ risk stratification categories were dichotomized into test-negative (low-risk: 0–40) and test-positive (moderate-risk: 41–70 and high-risk: 71–100) groups, consistent with conventional diagnostic test evaluation frameworks.

Exact binomial 95% confidence intervals were calculated for all proportions. Chi-square tests were used to assess differences in referral outcomes across study sites, with statistical significance defined a priori as a two-sided p-value < 0.05.

Breast AI™ (Med AI Sol Pty Ltd, South Africa) is a SAPHRA-registered Type A medical device designed as an adjunctive ultrasound-based clinical decision-support platform for breast assessment. The system was integrated with handheld Clarius™ wireless ultrasound probes and deployed within routine clinical workflows across participating healthcare sites. During ultrasound acquisition, imaging data were processed in real time through a proprietary AI-based analytical framework developed using previously validated breast ultrasound datasets comprising more than 40,000 histologically confirmed breast cancer cases.

The Breast AI™ platform generates a probabilistic malignancy risk score ranging from 0% to 100% based on ultrasound-derived imaging characteristics associated with lesion morphology and malignancy probability. Generated outputs were categorised into predefined low-risk (0–40%), moderate-risk (41–55%), and high-risk (56–100%) classifications to support structured clinical risk stratification. The system does not directly identify tumour cells or establish histopathological diagnoses, but rather provides an imaging-based probability assessment intended to complement clinical interpretation.

AI-generated outputs were displayed through an Android-based mobile interface accessible to registered healthcare professionals at the point of care. Breast AI™ assessments were performed concurrently with standard ultrasound examinations and clinical breast assessment workflows. Importantly, AI-derived classifications did not override clinician judgement or independently determine patient management decisions. All referrals, biopsies, follow-up recommendations, and downstream clinical decisions remained the responsibility of the attending healthcare professionals according to site-specific clinical protocols and standard diagnostic pathways.

Prior validation studies reported a diagnostic accuracy of 97.6% for the Breast AI™ system under development and testing conditions. However, the present study was designed to evaluate observational real-world implementation performance across heterogeneous clinical environments rather than to independently validate standalone diagnostic superiority over conventional imaging assessment pathways [16].

## Results

A total of 1,129 female patients were included in the study. The cohort had a mean age of 52.4 ± 11.8 years (range: 30–80 years) and comprised both premenopausal and postmenopausal women assessed across five heterogeneous healthcare sites between 1 April 2023 and 30 April 2025.

Across the 1,129 clinical assessments, 417 patients underwent referral for further diagnostic evaluation, of whom 405 underwent histopathological confirmation of malignancy on histopathological assessment. The proportion of referred patients subsequently diagnosed with malignancy was 97.1%. Site-level referral-associated malignancy rates ranged from 95.6% at the tertiary referral hospital (Site 5) to 100% at both the private diagnostic clinic (Site 2) and the community-based clinic setting (Site 1) (Table 1). Referral patterns remained stable across heterogeneous healthcare settings within the observational AI-supported assessment pathway. However, these findings should be interpreted as descriptive observational outcomes and do not imply superiority over standard diagnostic workflows or causal improvement in referral performance.

**Table 1:** STARD-style participant flow diagram demonstrating patient enrolment, Breast AI™ risk stratification, referral pathways, histopathological verification, and final analytic cohorts.

Study Stage	Number of Patients (n)	Description
Patients assessed for eligibility	1,129	Women presenting for routine or symptom-driven breast assessment across five participating healthcare sites
Excluded prior to final analysis	Not separately quantified*	Exclusions included incomplete imaging datasets, prior history of breast cancer, or ongoing primary endocrine therapy
Included in final analytic cohort	1,129	Patients undergoing Breast AI™-supported ultrasound assessment
Classified as Low Risk (0–40%)	508	Test-negative category for diagnostic accuracy analysis
Classified as Moderate Risk (41–55%)	211	Test-positive category
Classified as High Risk (56–100%)	410	Test-positive category
Total referred for further diagnostic evaluation	417	Referral based on routine clinical decision-making and imaging assessment
Histopathologically confirmed malignancy	405	Final verified malignant diagnoses
No histopathological malignancy identified	724	Includes non-referred low-risk patients and referred patients without confirmed malignancy
High-risk patients with confirmed malignancy	392	Histopathologically confirmed cancer within high-risk category
Low-risk patients with confirmed malignancy (false negatives)	3	Histopathologically confirmed malignancy despite low-risk classification
Final cohort included in diagnostic performance analysis	1,129	Included in sensitivity, specificity, PPV, and NPV calculations

**Fig 1:**
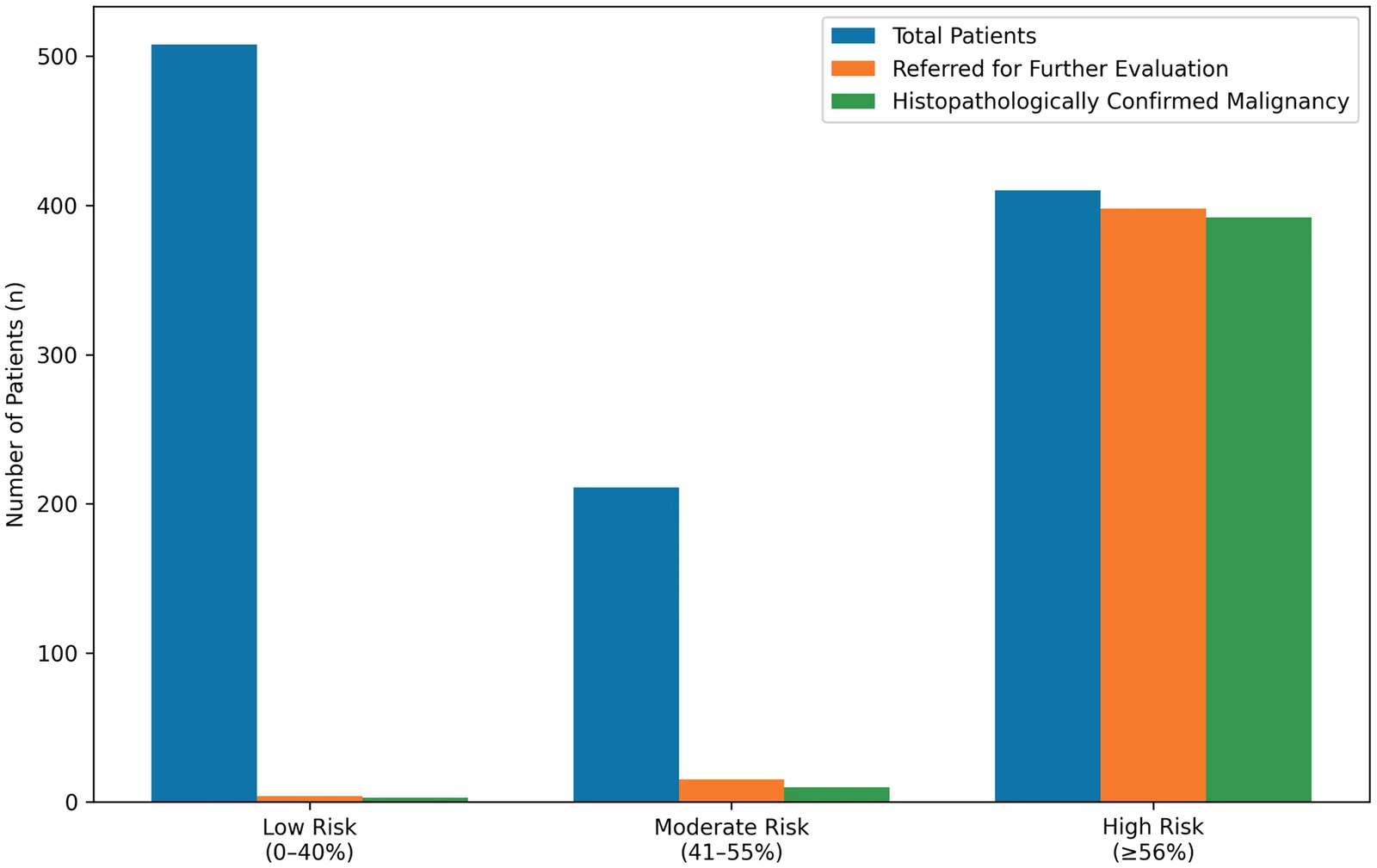
Breast AI™ risk stratification and downstream clinical outcomes. The figure illustrates the distribution of patients across predefined Breast AI™ risk categories and associated downstream clinical outcomes. Bars represent the total number of patients classified within each risk category, the number referred for further diagnostic evaluation, and the number with histopathologically confirmed malignancy. Increasing AI-derived risk classification was associated with progressively higher referral rates and malignancy confirmation rates. The figure provides a descriptive summary of patient flow following AI-supported risk stratification within the observational clinical pathway and does not imply comparative diagnostic superiority or causal inference.

Variability in referral proportions across participating sites likely reflected differences in healthcare setting type, patient demographics, referral pathways, disease prevalence, and local diagnostic infrastructure. Chi-square analysis demonstrated no statistically significant difference in referral-associated malignancy outcomes across participating sites (χ² = 1.67, p = 0.795), supporting relative consistency in observed referral-associated diagnostic yield despite heterogeneous healthcare environments. Breast AI™-supported ultrasound assessment was operationally integrated across community, rural, tertiary, and private-sector environments without major disruption to routine clinical workflows. Patient flow following AI-derived risk stratification and downstream referral outcomes is summarised in Table 2 and Fig 1.

**Table 2:** Referral and Histopathological Outcomes Among Patients Undergoing Further Diagnostic Evaluation Across Participating Healthcare Sites.

Site	Healthcare Setting	Patients Undergoing Further Diagnostic Evaluation (n)	Histopathologically Confirmed Malignancy (n)	Referral-Associated Malignancy Rate (%)
Site 1	Community-based clinic	1	1	100.0
Site 2	Private diagnostic clinic	14	14	100.0
Site 3	High-volume urban breast centre	259	253	97.7
Site 4	Rural district hospital	28	27	96.4
Site 5	Tertiary referral hospital	115	110	95.7
**Total**		**417**	**405**	**97.1**

Among patients who underwent histopathological verification, the median interval between Breast AI™-supported ultrasound assessment and definitive histopathological diagnosis was 18 days (interquartile range [IQR]: 10–32 days) Variability in diagnostic interval likely reflected differences in referral pathways, biopsy scheduling processes, healthcare resource availability, specialist access, and institutional workflow infrastructure across participating healthcare sites.

Analysis of Breast AI™ risk stratification categories demonstrated a clear relationship between increasing AI-derived risk classification, referral patterns, and downstream malignancy confirmation rates (Table 3). Patients classified within the high-risk category demonstrated substantially higher referral rates and histopathologically confirmed malignancy rates than those classified within the low-risk category. Specifically, 97.1% of patients categorised as high risk underwent further diagnostic investigation, with malignancy confirmed in 95.6% of this subgroup. In contrast, only 0.8% of low-risk patients underwent referral for additional investigation, and confirmed malignancy was identified in 0.6% of low-risk classifications. These findings support the internal consistency of the Breast AI™ risk stratification framework within the observational clinical pathway evaluated in this study.

**Table 3:** Distribution of Breast AI™ risk categories, referral outcomes, and diagnoses.

Breast AI™ Risk Category	Risk Score Range (%)	Total Patients (n)	Referred for Further Evaluation (n)	Histopathologically Confirmed Malignancy (n)	No Confirmed Malignancy / No Histopathological Confirmation (n)	Referral Rate (%)	Malignancy Rate Within Category (%)
Low risk	0–40	508	4	3	505	0.8	0.6
Moderate risk	41–55	211	15	10	201	7.1	4.7
High risk	≥56	410	398	392	18	97.1	95.6
Total		1,129	417	405	724	36.9	35.9

**Fig 2:**
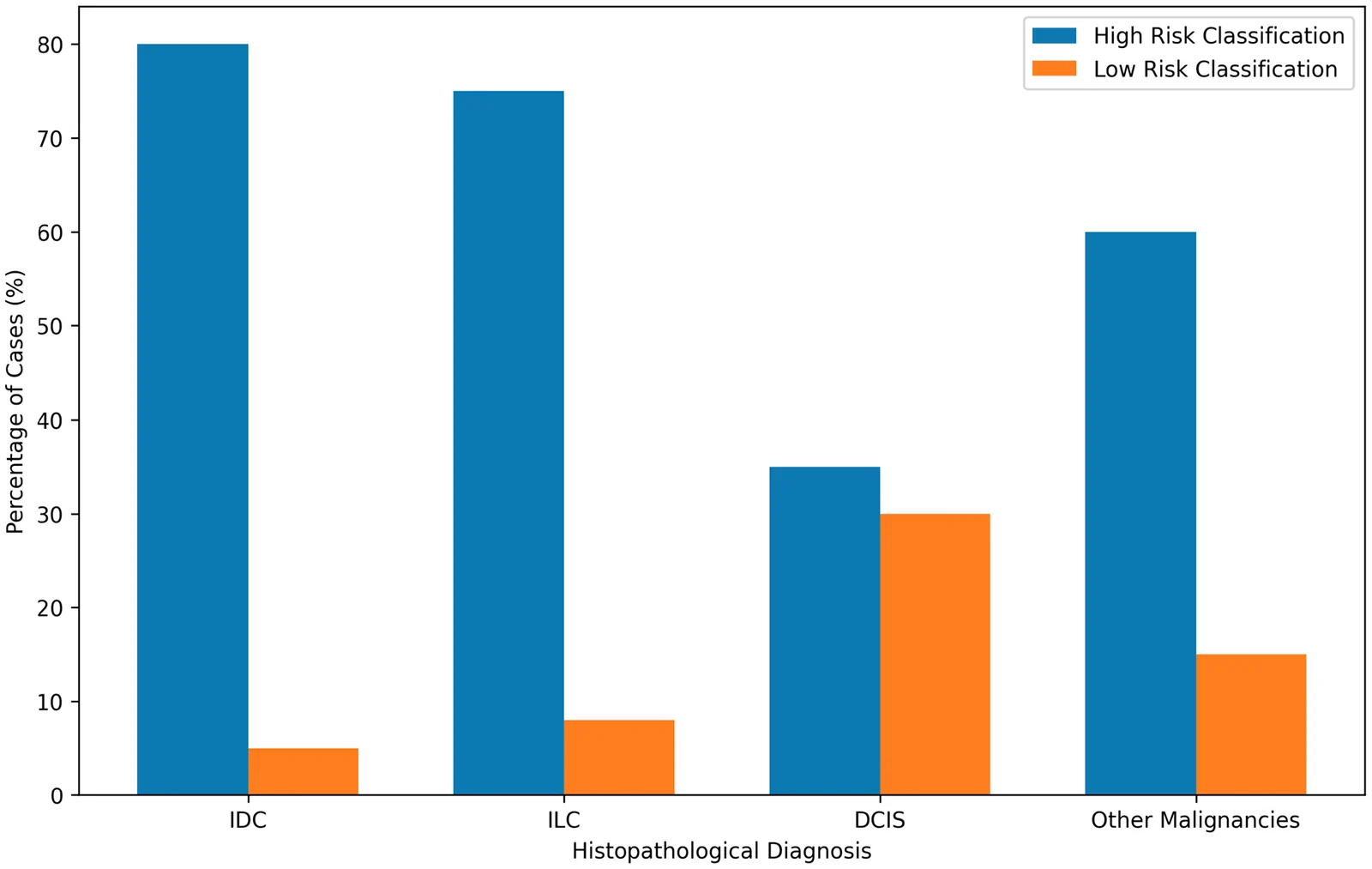
Distribution of Breast AI™ risk classifications across histopathological breast cancer subtypes. The figure illustrates the proportion of histopathologically confirmed breast cancer subtypes classified within higher- and lower-risk Breast AI™ categories. Invasive ductal carcinoma (IDC) and invasive lobular carcinoma (ILC) demonstrated greater representation within high-risk classifications, whereas a larger proportion of ductal carcinoma in situ (DCIS) lesions were classified within lower-risk categories. These findings suggest stronger AI-associated risk stratification performance for invasive malignancies while highlighting reduced sensitivity for certain early-stage non-invasive lesions. The figure is descriptive in nature and does not imply causal inference or comparative superiority relative to conventional diagnostic assessment pathways.

The pathological distribution of confirmed malignancies demonstrated that invasive ductal carcinoma (IDC) accounted for the largest proportion of cases (45.0%, n = 182), followed by ductal carcinoma in situ (DCIS) (35.1%, n = 142), invasive lobular carcinoma (ILC) (10.1%, n = 41), and other malignant subtypes (9.9%, n = 40). Higher Breast AI™ risk classifications were more frequently associated with invasive malignancies, with 80% of IDC cases and 75% of ILC cases categorised within the high-risk group. In contrast, approximately 30% of DCIS lesions were classified within the low-risk category, highlighting reduced sensitivity for certain early-stage non-invasive malignancies and identifying an important area for future algorithm refinement (Fig 2).

Diagnostic performance analysis demonstrated a sensitivity of 99.3% (95% CI: 97.8–99.8), specificity of 69.8% (95% CI: 66.3–73.1), positive predictive value (PPV) of 64.7% (95% CI: 61.2–68.0), and negative predictive value (NPV) of 99.4% (95% CI: 98.3–99.8) (Table 4). The high observed NPV suggests that patients classified within the low-risk category were unlikely to demonstrate histologically confirmed malignancy within the available verification cohort. However, the comparatively lower PPV and specificity reflect the trade-off between maintaining high sensitivity and the potential for increased referral burden and false-positive classifications within real-world breast assessment pathways. Because histopathological verification was not uniformly obtained across all risk categories, these findings should be interpreted cautiously within the context of differential verification bias inherent to observational diagnostic studies.

**Table 4:** Diagnostic Performance of Breast AI™ Risk Stratification Using Dichotomised Risk Categories.

Diagnostic Outcome	Malignancy Present	Malignancy Absent	Total
Test-positive (Moderate/High Risk)	402 (TP)	219 (FP)	621
Test-negative (Low Risk)	3 (FN)	505 (TN)	508
**Total**	**405**	**724**	**1,129**
Metric	Value % (95% CI)		
Sensitivity	99.3% (97.8–99.8)		
Specificity	69.8% (66.3–73.1)		
Positive Predictive Value (PPV)	64.7% (61.2–68.0)		

The observed PPV reflects the proportion of patients classified as test-positive who were subsequently confirmed to have malignancy following further diagnostic evaluation. While the high sensitivity and negative predictive value observed in the present study suggest that the AI-supported pathway was effective in identifying patients unlikely to harbour malignancy, the comparatively lower specificity and PPV indicate the potential for increased referral burden and additional downstream investigations among patients without confirmed cancer. This trade-off between maximising sensitivity and limiting unnecessary referrals is well recognised in breast cancer assessment pathways, particularly within screening and triage-oriented diagnostic frameworks.

Within resource-constrained healthcare settings, maintaining high sensitivity may be clinically advantageous because delayed diagnosis and missed malignancies are associated with poorer outcomes and advanced-stage presentation. However, increased false-positive classifications may also contribute to additional imaging investigations, biopsy procedures, patient anxiety, and healthcare resource utilisation. Consequently, interpretation of PPV should be balanced against the clinical objective of minimising missed invasive malignancies, particularly within heterogeneous populations where access to specialist breast imaging services may already be limited.

The high observed NPV suggests that patients classified within the low-risk category were unlikely to demonstrate histologically confirmed malignancy within the available verification cohort. However, because histopathological confirmation was primarily obtained in patients referred for further investigation, these findings should be interpreted cautiously within the context of potential differential verification bias inherent to observational diagnostic studies. In addition, long-term interval cancer follow-up was not available for all low-risk patients, limiting definitive interpretation of the observed NPV in broader population-level screening settings. Because the study cohort included a relatively high proportion of symptomatic and referred patients, spectrum bias may additionally influence the observed diagnostic performance metrics relative to population-based screening cohorts.

## Discussion

The findings of this prospective multi-site observational study demonstrate that Breast AI™-supported ultrasound assessment achieved high negative predictive performance and reproducible risk stratification across heterogeneous healthcare environments. The observed diagnostic metrics are broadly comparable to previously reported outcomes for AI-assisted breast imaging systems described in the literature [2,5,6]. Previous studies evaluating AI-supported breast imaging have reported pooled sensitivities and specificities ranging between approximately 85% and 90%, although substantial variability exists across imaging modalities, study populations, and disease verification methodologies [2,5,6]. Within the present study, Breast AI™ demonstrated high sensitivity and negative predictive value when predefined risk categories were dichotomised into test-negative and test-positive groups.

Importantly, these findings should be interpreted within the methodological constraints of the observational study design and the presence of differential disease verification. Histopathological confirmation was primarily obtained in patients referred for additional diagnostic investigation, while the majority of low-risk patients did not undergo systematic tissue verification. As described by Alonzo and Pepe, selective verification of disease status may introduce substantial bias into diagnostic accuracy estimation, particularly for sensitivity and negative predictive value calculations within screening and triage studies [19]. Consequently, the high observed negative predictive value should be interpreted cautiously, as interval cancers among low-risk patients could not be comprehensively excluded in the absence of long-term surveillance follow-up.

The current study therefore does not establish superiority over radiologist-led assessment pathways or conventional imaging workflows, but rather describes diagnostic performance observed within an AI-supported real-world implementation setting. Furthermore, because histopathological confirmation was preferentially performed in clinically suspicious or referred cases, the reported diagnostic metrics may overestimate true population-level performance characteristics. These limitations are particularly relevant when interpreting the low false-negative rate observed within the present cohort.

The multi-site design nevertheless represents an important strength of the study. Breast AI™ was integrated across community-based clinics, rural healthcare facilities, tertiary referral centres, and private-sector imaging environments encompassing variable patient populations, referral pathways, and diagnostic infrastructure. Despite these operational differences, referral-associated malignancy rates remained relatively stable across participating sites, and no statistically significant site-level differences were identified on chi-square analysis. These findings suggest that AI-supported ultrasound risk stratification may be feasibly incorporated into diverse healthcare settings, including resource-constrained environments where specialist breast imaging services are limited.

Implementation of AI-supported imaging systems across heterogeneous clinical environments additionally requires consideration of several operational and infrastructural factors. Variability in ultrasound equipment, image acquisition technique, operator training, quality assurance processes, calibration procedures, internet connectivity, and integration with existing clinical workflows may all influence real-world AI performance and reproducibility. In the present study, Breast AI™ was deployed using standardised handheld ultrasound hardware and predefined risk stratification protocols across participating sites to reduce inter-site variability. Nevertheless, differences in operator experience and institutional workflow processes remain potential sources of implementation variability. As highlighted by Kotter and Ranschaert, successful clinical integration of AI systems requires not only technical performance validation, but also structured governance frameworks, clinician engagement, workflow integration strategies, ongoing quality assurance, and user training to ensure safe and sustainable adoption within routine practice [20].

The relevance of such implementation pathways is particularly important within LMIC settings, where delayed diagnosis, limited mammographic infrastructure, and shortages of trained radiology personnel continue to contribute to poor breast cancer outcomes [8–12,17,18]. Previous studies from sub-Saharan Africa and other LMIC regions have demonstrated the potential value of ultrasound-based and clinical assessment pathways for facilitating earlier detection and clinical downstaging [8,9,11]. The portability of handheld ultrasound systems, combined with AI-supported risk stratification, may therefore represent a potentially scalable adjunctive approach for supporting breast assessment workflows in under-resourced environments. However, the present study did not directly evaluate healthcare efficiency, cost-effectiveness, workflow throughput, reporting time, or downstream economic outcomes, and any implications regarding operational benefit should therefore be interpreted cautiously and regarded as exploratory [17–20].

An additional finding of clinical importance was the relationship between Breast AI™ risk classification and histopathological subtype distribution. Higher-risk classifications were more frequently associated with invasive malignancies, particularly invasive ductal carcinoma and invasive lobular carcinoma. In contrast, approximately 30% of ductal carcinoma in situ (DCIS) lesions were classified within the low-risk category. This observation highlights a recognised limitation of both AI-supported and conventional imaging assessment approaches, namely reduced sensitivity for subtle early-stage or non-invasive lesions with limited overt morphological distortion [3,4]. The findings suggest that further algorithm refinement may be necessary to improve detection of early non-invasive malignancies, particularly within screening or surveillance contexts.

The study additionally demonstrated a relationship between AI-derived risk categories, referral patterns, and downstream histopathological outcomes. Patients classified within the high-risk category demonstrated substantially higher referral and malignancy confirmation rates than those classified as low risk. Although these findings support the internal consistency of the AI-derived stratification framework, caution is required when interpreting positive predictive value and referral-associated malignancy rates, as these outcomes are influenced by underlying disease prevalence, referral thresholds, and selective verification practices within the participating clinical environments.

The proposed BI-AI RADS framework was developed as a conceptual approach to facilitate structured integration of AI-derived risk outputs into clinical assessment pathways (Table 5). The framework aims to standardise communication of AI-supported risk categories and align algorithm-generated outputs with clinically interpretable management pathways analogous to existing BI-RADS reporting principles. Such structured reporting approaches may support multidisciplinary communication, educational standardisation, and integration of AI-supported assessment into broader diagnostic workflows. Nevertheless, the proposed framework remains preliminary and requires external validation across larger and more diverse populations before broader implementation can be recommended.

**Table 5:** Proposed Framework for B-AI RADS System.

BI-AI RADS Category	Scores	Clinical Implication	Literature Support
Low Risk (BI-AI RADS 1-2)	0-40%	Likely benign findings.	Studies indicate low AI scores correlate with minimal structural distortion and no malignant features [1,2].
		Routine follow-up or short-term monitoring may suffice.	
Moderate Risk (BI-AI RADS 3-4A)	41-55%	Suspicious findings requiring additional imaging or biopsy.	Moderate AI scores correspond to findings like atypical ductal hyperplasia or DCIS [3].
High Risk (BI-AI RADS 4B-5)	56-100%	Highly suspicious for malignancy, warranting immediate biopsy or intervention.	High AI scores align with invasive cancers (e.g., IDC, ILC), consistent with human radiologist predictions [4,5].

Several important limitations should be acknowledged. First, the study was conducted without a parallel comparator cohort undergoing standard radiologist-led assessment alone, limiting the ability to determine whether Breast AI™ improves diagnostic performance relative to existing clinical workflows. Second, differential verification bias may have substantially influenced observed diagnostic accuracy estimates because histopathological confirmation was not uniformly obtained across all risk categories. Third, long-term follow-up data were not available for all patients classified as low risk, limiting assessment of interval cancers and restricting definitive interpretation of the observed negative predictive value. Fourth, the study did not formally assess workflow efficiency metrics, economic outcomes, radiologist workload, biopsy burden, or patient-centred outcomes. Finally, variability in operator experience, imaging acquisition, site-level infrastructure, and local referral practices may affect generalisability despite the multi-site design.

Overall, the findings support the feasibility of integrating AI-supported ultrasound risk stratification into heterogeneous clinical environments and contribute prospective observational data regarding implementation within resource-variable healthcare settings. Further comparative studies incorporating standardised verification pathways, longitudinal interval cancer follow-up, subgroup stratification analyses, and formal health-economic evaluation will be necessary to more fully define the clinical role of AI-supported breast ultrasound assessment in routine practice.

## Conclusion

This prospective multi-site observational study demonstrated that Breast AI™-supported ultrasound assessment achieved high observed negative predictive value and reproducible risk stratification across heterogeneous healthcare settings. Breast AI™ categorised patients into predefined low-, moderate-, and high-risk groups based on algorithm-derived probability thresholds, with higher-risk classifications more frequently associated with invasive malignancies.

However, the study was not designed to directly compare AI-supported assessment pathways with radiologist-only workflows or pre-implementation diagnostic models. In addition, the absence of systematic histopathological verification among low-risk patients and the lack of long-term interval cancer follow-up introduce uncertainty regarding the true population-level sensitivity and negative predictive value of the system. Differential verification bias may therefore have influenced the observed diagnostic performance metrics.

The findings support the feasibility of integrating AI-supported ultrasound assessment into diverse and resource-variable healthcare environments, particularly in settings where access to specialist breast imaging services may be limited. Nevertheless, the present study did not directly evaluate workflow efficiency, reporting time, healthcare costs, radiologist workload, or downstream economic outcomes. Consequently, any implications regarding operational benefit or healthcare efficiency should be interpreted cautiously and regarded as exploratory.

Further prospective comparative studies incorporating standardised verification protocols, longitudinal interval cancer follow-up, external validation cohorts, and formal health-economic analyses will be necessary to establish the broader clinical utility and implementation value of AI-supported breast ultrasound assessment pathways.
