# Supplementary material for: Multi-site observational evaluation of Breast AI™-supported ultrasound risk stratification in breast cancer assessment pathways
Source: PLoS One. 2026 Sep 15;21(9):e0357975. doi: 10.1371/journal.pone.0357975 (PMC13577415; doi:10.1371/journal.pone.0357975)
Supplement: Peer review history

# Peer review: Decision Letter 0

**DOI:** 10.1371/journal.pone.0357975.r001

**Authors:** Elingarami Sauli

13 Oct 2025

Dear Dr. Kathryn,

Thank you for submitting your manuscript to PLOS ONE. After careful consideration, we feel that it has merit but does not fully meet PLOS ONE’s publication criteria as it currently stands. Therefore, we invite you to submit a revised version of the manuscript that addresses the points raised during the review process.

==============================

The authors should improve the submission, especially on clear elaboration of results from conducted statistical analysis, clarity on metrics used for specificity calculation, and also general improvement of discussion of obtained results.

==============================

Please submit your revised manuscript by  Nov 27 2025 11:59PM. If you will need more time than this to complete your revisions, please reply to this message or contact the journal office at plosone@plos.org. When you're ready to submit your revision, log on to https://www.editorialmanager.com/pone/ and select the 'Submissions Needing Revision' folder to locate your manuscript file.

- A rebuttal letter that responds to each point raised by the academic editor and reviewer(s). You should upload this letter as a separate file labeled 'Response to Reviewers'.
- A marked-up copy of your manuscript that highlights changes made to the original version. You should upload this as a separate file labeled 'Revised Manuscript with Track Changes'.
- An unmarked version of your revised paper without tracked changes. You should upload this as a separate file labeled 'Manuscript'.

If you would like to make changes to your financial disclosure, please include your updated statement in your cover letter. Guidelines for resubmitting your figure files are available below the reviewer comments at the end of this letter.

If applicable, we recommend that you deposit your laboratory protocols in protocols.io to enhance the reproducibility of your results. Protocols.io assigns your protocol its own identifier (DOI) so that it can be cited independently in the future. For instructions see: https://journals.plos.org/plosone/s/submission-guidelines#loc-laboratory-protocols. Additionally, PLOS ONE offers an option for publishing peer-reviewed Lab Protocol articles, which describe protocols hosted on protocols.io. Read more information on sharing protocols at https://plos.org/protocols?utm_medium=editorial-email&utm_source=authorletters&utm_campaign=protocols.

We look forward to receiving your revised manuscript.

Kind regards,

Elingarami Sauli, PhD

Academic Editor

PLOS ONE

Journal Requirements:

When submitting your revision, we need you to address these additional requirements.

1. Please ensure that your manuscript meets PLOS ONE's style requirements, including those for file naming. The PLOS ONE style templates can be found at

https://journals.plos.org/plosone/s/file?id=wjVg/PLOSOne_formatting_sample_main_body.pdf and

https://journals.plos.org/plosone/s/file?id=ba62/PLOSOne_formatting_sample_title_authors_affiliations.pdf

2. Please include a complete copy of PLOS’ questionnaire on inclusivity in global research in your revised manuscript. Our policy for research in this area aims to improve transparency in the reporting of research performed outside of researchers’ own country or community. The policy applies to researchers who have travelled to a different country to conduct research, research with Indigenous populations or their lands, and research on cultural artefacts. The questionnaire can also be requested at the journal’s discretion for any other submissions, even if these conditions are not met. Please find more information on the policy and a link to download a blank copy of the questionnaire here: https://journals.plos.org/plosone/s/best-practices-in-research-reporting. Please upload a completed version of your questionnaire as Supporting Information when you resubmit your manuscript.

3. When completing the data availability statement of the submission form, you indicated that you will make your data available on acceptance. We strongly recommend all authors decide on a data sharing plan before acceptance, as the process can be lengthy and hold up publication timelines. Please note that, though access restrictions are acceptable now, your entire data will need to be made freely accessible if your manuscript is accepted for publication. This policy applies to all data except where public deposition would breach compliance with the protocol approved by your research ethics board. If you are unable to adhere to our open data policy, please kindly revise your statement to explain your reasoning and we will seek the editor's input on an exemption. Please be assured that, once you have provided your new statement, the assessment of your exemption will not hold up the peer review process.

4. Please ensure that you include a title page within your main document. You should list all authors and all affiliations as per our author instructions and clearly indicate the corresponding author.

5. Please include your full ethics statement in the ‘Methods’ section of your manuscript file. In your statement, please include the full name of the IRB or ethics committee who approved or waived your study, as well as whether or not you obtained informed written or verbal consent. If consent was waived for your study, please include this information in your statement as well.

6. Please include captions for your Supporting Information files at the end of your manuscript, and update any in-text citations to match accordingly. Please see our Supporting Information guidelines for more information: http://journals.plos.org/plosone/s/supporting-information.

7. If the reviewer comments include a recommendation to cite specific previously published works, please review and evaluate these publications to determine whether they are relevant and should be cited. There is no requirement to cite these works unless the editor has indicated otherwise.

[Note: HTML markup is below. Please do not edit.]

Reviewers' comments:

Reviewer's Responses to Questions

**Comments to the Author**

1. Is the manuscript technically sound, and do the data support the conclusions?

Reviewer #1: No

Reviewer #2: Partly

**********

2. Has the statistical analysis been performed appropriately and rigorously?

Reviewer #1: No

Reviewer #2: Yes

**********

3. Have the authors made all data underlying the findings in their manuscript fully available?

The PLOS Data policy

Reviewer #1: Yes

Reviewer #2: Yes

**********

4. Is the manuscript presented in an intelligible fashion and written in standard English?

Reviewer #1: Yes

Reviewer #2: Yes

**********

Reviewer #1: The authors have presented an observational study to assess the diagnostic accuracy of the Breast AI system, an AI tool designed to support the detection of breast cancer, in five sites. The manuscript is well written but some improvements are required to enhance clarity and reader-interpretability. I therefore have these comments for the authors:

1. Study design section – the study is reported to be an observational study. In such studies, interventions do not inform care, but the Methods say that patients received "Breast AI™-supported ultrasound clinical assessments", suggesting Breast AI did inform care. Could you please clarify whether or not Breast AI was used to inform care in this study and whether this was or was not an observational study?

2. Study design section – it says, “Participants received standard care, including clinical breast examinations (CBE), specialist consultations, and concurrent Breast AI™-supported ultrasound clinical assessments”. As above, this sentence reads as if Breast AI is standard of care in these sites. Could you please confirm if this true?

3. Study design section – it says, “The cohort had a mean age…” This sentence is results, not methods, so should be in the Results section.

4. Study design section – the paragraph starting “Data collection involved…” is not entirely study design, as it includes analysis details, such as the metrics used. I would suggest moving the analysis details to a new section with the heading “Data analysis”.

5. Study design section – it says the NPV was assessed but there are no corresponding results in the Results section.

6. Study design section – the diagnostic accuracy of the Breast AI was assessed, but Breast AI produces three risk groups (low, moderate, and high). Diagnostic accuracy measures work when there are two risk groups, not three. Could you please clarify whether the three risk groups were converted into two risk groups to assess diagnostic accuracy and how?

7. Study design section – could the authors please specify the significance level used for chi-squared testing.

8. Study design section – it says chi-squared tests were performed but there are no corresponding results in the Results section.

9. Results section – there are no in-text citations to tables and figures. Please could you add these?

10. Results section – can you be confident that Breast AI was the reason for the high referral accuracy? To assess this, you would need a comparator group, such as people who were referred based on CBE or specialist consultation alone and then compare the referral in this group to the group including Breast AI supported ultrasound. I do not believe you can say that your findings validate the scalability and precision of Breast AI in enhancing the referral accuracy without doing such a comparison.

11. Results section – specificity is the % of cancer-free patients who were correctly identified as low risk. However, you have a moderate risk group too, so how was this group considered in the calculation of specificity (and the other metrics)?

12. Results section – the third paragraph focuses primarily on diagnostic accuracy metrics in the high-risk group, but we need to know what happens in the moderate- and low-risk groups in order to get a better picture of how well Breast AI works. It would be helpful if the authors could please include a table that shows the number of people classified as low, moderate, or high risk out of the total 1129, then for each risk group, the number referred and diagnosed, and the diagnostic accuracy.

13. Results section – the last four paragraphs of the Results are a comparison with existing literature and summary of findings so should be in the Discussion section, not Results section.

14. Results section – I am wondering if there a partial verification bias in the diagnostic accuracy. This bias happens when a key group is missing from the diagnostic accuracy calculation. For example, of the 1129 patients in the study, presumably the high-risk patients received further investigation. If this were true, we would only expect to see diagnoses among referred patients. The fact that low-risk patients are not referred means we do not pick up cancer in these individuals, so it would appear that Breast AI has good diagnostic accuracy but this would be biased, as not all patients are included in the calculation. As mentioned in another comment, it would be helpful to have a table that shows how many of the 1129 patients were considered low/moderate/high and how many in each risk group were referred and diagnosed. This would help clarify the patient flow and allow readers to get a better sense of how well Breast AI works.

15. Discussion – throughout, you mention that Breast AI is very good. However, I am struggling to see the added benefit of it. Do you know what referral accuracy was like before Breast AI was implemented? We would need to see those in order to assess the benefit of Breast AI in these cohorts/sites.

16. Discussion – in paragraph 3, you say, “Breast AI™ demonstrated superior consistency and objectivity, maintaining high referral accuracy across diverse operational contexts.” I believe you can only say that Breast AI demonstrated superiority if you investigated this. It seems like you did not though, as there are no findings from a comparator group where Breast AI was not used. Could you please clarify?

17. Discussion – in paragraph 4, you say, “Breast AI™ successfully categorized patients…” It is unclear what “successfully” means. I personally would not say "successfully" because there are many study design and methods-related clarifications that are needed before we can say it is successful.

18. Discussion – in paragraph 4, you say, “While Breast AI™ outperformed radiologists in stratifying invasive cancers, the system's challenges…” Again, I do not think you can say that Breast AI outperformed radiologists in stratifying invasive cases without doing a radiologist vs radiologist+Breast AI comparison. If such an analysis was done, please could you add those results or a reference to them to support such statements?

19. Discussion – in paragraph 5, you say, “Breast AI™ also demonstrated significant workflow efficiencies by pre- clinical assessment cases and identifying those warranting radiologist review. This reduced radiologist workload, allowing them to focus on complex or ambiguous cases.” As above, this seems speculative, as there are no findings reported to support this statement.

20. Discussion – in paragraph 6, you say, “Breast AI™ consistently demonstrated comparable or superior performance to radiologists in diagnostic accuracy, referral precision, and workflow efficiency, particularly in detecting high- risk invasive cancers.” Where are these results? It would be helpful to include them.

21. Table 1 – I would not say that the findings demonstrate scalability, because, as I understand this study design to be, we do not know what happens in people who are not referred. It could be that many of them had undiagnosed cancer so should have been referred. So I would suggest rewording this.

22. Table 2 – the sample sizes are quite low for some sites. Can you please add 95% confidence intervals for these proportions? They can be calculated using the binomial confidence interval estimator.

23. Figure 1 – the legend says, “It highlights consistency in AI performance and site-specific variations in referral accuracy.” However, Figure 1 is not broken down by site, so I would suggest removing this sentence.

24. Figure 1 – could you please label the diagnosis bars by add the % diagnosed so we can see how diagnosis trends have changed in relation to referral rates?

25. Figure 2 – could you please clarify what the % is? This is in the middle of two bars so it is unclear whether this is the % referred or % diagnosed.

26. Figure 3 – is my understanding correct that a risk prediction score was derived for all 1129 patients in the study? If so, could you give these %s for those who were not diagnosed? This could potentially go in the additional table I have requested.

Reviewer #2: The abstract is too long with unnecessary information; it should be a concise summary of the paper. Some abbreviations need to be spelling at 1st use (eg SPSS, SAPHRA). The results section contains much discussion, it should be only the results. The technology and the actual assessment carried out is not well explained. I still do not known what this specific AI system does. More background on the tool, development, reliability, its use in other setting etc, etc should be included.

**********

what does this mean?). If published, this will include your full peer review and any attached files.

If you choose “no”, your identity will remain anonymous but your review may still be made public.

**Do you want your identity to be public for this peer review?**  For information about this choice, including consent withdrawal, please see our Privacy Policy

Reviewer #1: No

Reviewer #2: No

**********

[NOTE: If reviewer comments were submitted as an attachment file, they will be attached to this email and accessible via the submission site. Please log into your account, locate the manuscript record, and check for the action link "View Attachments". If this link does not appear, there are no attachment files.]

While revising your submission, please upload your figure files to the Preflight Analysis and Conversion Engine (PACE) digital diagnostic tool, https://pacev2.apexcovantage.com/. PACE helps ensure that figures meet PLOS requirements. To use PACE, you must first register as a user. Registration is free. Then, login and navigate to the UPLOAD tab, where you will find detailed instructions on how to use the tool. If you encounter any issues or have any questions when using PACE, please email PLOS at figures@plos.org. Please note that Supporting Information files do not need this step.

# Author comment: Author response to Decision Letter 1

**DOI:** 10.1371/journal.pone.0357975.r002

3 Feb 2026

Reviewer #1 Author Response Manuscript Change

1. Study design section – the study is reported to be an observational study. In such studies, interventions do not inform care, but the Methods say that patients received "Breast AI™-supported ultrasound clinical assessments", suggesting Breast AI did inform care. Could you please clarify whether or not Breast AI was used to inform care in this study and whether this was or was not an observational study? We clarify that Breast AI™ did not determine definitive clinical management. It functioned as a decision-support and triage tool, with final referral decisions made by clinicians according to site protocols. The study therefore remains a prospective observational cohort study, not an interventional trial. Study Design clarified; wording revised

2. Study design section – it says, “Participants received standard care, including clinical breast examinations (CBE), specialist consultations, and concurrent Breast AI™-supported ultrasound clinical assessments”. As above, this sentence reads as if Breast AI is standard of care in these sites. Could you please confirm if this true? We agree the wording was misleading. Breast AI™ was adjunctive to standard care, not itself standard of care. Text has been corrected accordingly. Study Design paragraph revised

3. Study design section – it says, “The cohort had a mean age…” This sentence is results, not methods, so should be in the Results section. Accepted. Demographic characteristics have been relocated to the Results section. Moved to Results

4. Study design section – the paragraph starting “Data collection involved…” is not entirely study design, as it includes analysis details, such as the metrics used. I would suggest moving the analysis details to a new section with the heading “Data analysis”.

Accepted. A new “Statistical Analysis” subsection has been created. New subsection added

5. Study design section – it says the NPV was assessed but there are no corresponding results in the Results section. Accepted. NPV values are now reported with corresponding confidence intervals. Results expanded

6. Study design section – the diagnostic accuracy of the Breast AI was assessed, but Breast AI produces three risk groups (low, moderate, and high). Diagnostic accuracy measures work when there are two risk groups, not three. Could you please clarify whether the three risk groups were converted into two risk groups to assess diagnostic accuracy and how? Clarified. For diagnostic accuracy calculations, moderate- and high-risk categories were combined into a single “test-positive” group, consistent with standard diagnostic test methodology. This is now explicitly stated. Methods clarified

7. Study design section – could the authors please specify the significance level used for chi-squared testing.

Significance threshold of α = 0.05 (two-sided) has been specified. Methods updated

8. Study design section – it says chi-squared tests were performed but there are no corresponding results in the Results section.

Accepted. Site-level chi-square results are now reported where applicable. Results updated

9. Results section – there are no in-text citations to tables and figures. Please could you add these? Accepted. All tables and figures are now cited sequentially in text. Results revised

10. Results section – can you be confident that Breast AI was the reason for the high referral accuracy? To assess this, you would need a comparator group, such as people who were referred based on CBE or specialist consultation alone and then compare the referral in this group to the group including Breast AI supported ultrasound. I do not believe you can say that your findings validate the scalability and precision of Breast AI in enhancing the referral accuracy without doing such a comparison. Accepted. Language has been revised to avoid causal inference. Claims now reflect association, not validation of superiority or scalability. Results toned down

11. Results section – specificity is the % of cancer-free patients who were correctly identified as low risk. However, you have a moderate risk group too, so how was this group considered in the calculation of specificity (and the other metrics)? Clarified. Specificity was calculated by classifying low-risk as test-negative, and moderate/high as test-positive. This is now detailed in Methods. Methods clarified

12. Results section – the third paragraph focuses primarily on diagnostic accuracy metrics in the high-risk group, but we need to know what happens in the moderate- and low-risk groups in order to get a better picture of how well Breast AI works. It would be helpful if the authors could please include a table that shows the number of people classified as low, moderate, or high risk out of the total 1129, then for each risk group, the number referred and diagnosed, and the diagnostic accuracy. Accepted. A new table presents risk category distribution (n=1129), referrals, confirmed diagnoses, and diagnostic metrics per risk group. New Table added

13. Results section – the last four paragraphs of the Results are a comparison with existing literature and summary of findings so should be in the Discussion section, not Results section.

Accepted. Comparative literature and interpretive text moved to Discussion. Section reorganized

14. Results section – I am wondering if there a partial verification bias in the diagnostic accuracy. This bias happens when a key group is missing from the diagnostic accuracy calculation. For example, of the 1129 patients in the study, presumably the high-risk patients received further investigation. If this were true, we would only expect to see diagnoses among referred patients. The fact that low-risk patients are not referred means we do not pick up cancer in these individuals, so it would appear that Breast AI has good diagnostic accuracy but this would be biased, as not all patients are included in the calculation. As mentioned in another comment, it would be helpful to have a table that shows how many of the 1129 patients were considered low/moderate/high and how many in each risk group were referred and diagnosed. This would help clarify the patient flow and allow readers to get a better sense of how well Breast AI works. Acknowledged and explicitly discussed as a study limitation. Patient flow table added to improve transparency. Limitation added

15. Discussion – throughout, you mention that Breast AI is very good. However, I am struggling to see the added benefit of it. Do you know what referral accuracy was like before Breast AI was implemented? We would need to see those in order to assess the benefit of Breast AI in these cohorts/sites. Accepted. We clarify that no historical or parallel comparator cohortwas available; benefit claims are therefore restrained. Discussion revised

16. Discussion – in paragraph 3, you say, “Breast AI™ demonstrated superior consistency and objectivity, maintaining high referral accuracy across diverse operational contexts.” I believe you can only say that Breast AI demonstrated superiority if you investigated this. It seems like you did not though, as there are no findings from a comparator group where Breast AI was not used. Could you please clarify? Accepted. All claims of superiority have been replaced with “consistent performance” or “comparable outcomes”. Discussion revised

17. Discussion – in paragraph 4, you say, “Breast AI™ successfully categorized patients…” It is unclear what “successfully” means. I personally would not say "successfully" because there are many study design and methods-related clarifications that are needed before we can say it is successful. Accepted. Language softened to “categorized according to predefined risk thresholds”. Discussion revised

18. Discussion – in paragraph 4, you say, “While Breast AI™ outperformed radiologists in stratifying invasive cancers, the system's challenges…” Again, I do not think you can say that Breast AI outperformed radiologists in stratifying invasive cases without doing a radiologist vs radiologist+Breast AI comparison. If such an analysis was done, please could you add those results or a reference to them to support such statements?

Accepted. All direct AI-versus-radiologist performance claims removed. Discussion revised

19. Discussion – in paragraph 5, you say, “Breast AI™ also demonstrated significant workflow efficiencies by pre- clinical assessment cases and identifying those warranting radiologist review. This reduced radiologist workload, allowing them to focus on complex or ambiguous cases.” As above, this seems speculative, as there are no findings reported to support this statement. Accepted. Statements reframed as hypothesized operational implications, not demonstrated outcomes. Discussion revised

20. Discussion – in paragraph 6, you say, “Breast AI™ consistently demonstrated comparable or superior performance to radiologists in diagnostic accuracy, referral precision, and workflow efficiency, particularly in detecting high- risk invasive cancers.” Where are these results? It would be helpful to include them.

Accepted. Rewritten to reflect observed diagnostic metrics only, without comparative assertions. Discussion revised

21. Table 1 – I would not say that the findings demonstrate scalability, because, as I understand this study design to be, we do not know what happens in people who are not referred. It could be that many of them had undiagnosed cancer so should have been referred. So I would suggest rewording this.

Accepted. Reworded to describe multi-site feasibility, not scalability. Table legend revised

23. Figure 1 – the legend says, “It highlights consistency in AI performance and site-specific variations in referral accuracy.” However, Figure 1 is not broken down by site, so I would suggest removing this sentence. Accepted. Exact binomial 95% confidence intervals added to Table 2. Table updated

23. Figure 1 – the legend says, “It highlights consistency in AI performance and site-specific variations in referral accuracy.” However, Figure 1 is not broken down by site, so I would suggest removing this sentence. Accepted. Legend revised to remove site-specific inference. Figure legend revised

24. Figure 1 – could you please label the diagnosis bars by add the % diagnosed so we can see how diagnosis trends have changed in relation to referral rates?

Accepted. Percentages added to bars. Figure revised

25. Figure 2 – could you please clarify what the % is? This is in the middle of two bars so it is unclear whether this is the % referred or % diagnosed.

Accepted. Legend clarified to specify whether percentages represent referral or diagnosis rates. Figure legend revised

26. Figure 3 – is my understanding correct that a risk prediction score was derived for all 1129 patients in the study? If so, could you give these %s for those who were not diagnosed? This could potentially go in the additional table I have requested. Accepted. Included in new risk stratification table. New Table added

Reviewer #2: The abstract is too long with unnecessary information; it should be a concise summary of the paper. Some abbreviations need to be spelling at 1st use (eg SPSS, SAPHRA).

The results section contains much discussion, it should be only the results.

The technology and the actual assessment carried out is not well explained. I still do not known what this specific AI system does.

More background on the tool, development, reliability, its use in other setting etc, etc should be included.

Abstract p.1 1–3 Condensed; removed speculative claims

Study Design p.6 2–3 Clarified observational nature; AI not standard of care

Methods – Statistical Analysis p.8 1–2 New subsection; α=0.05; risk group dichotomization

Results – Demographics p.9 1 Age data moved from Methods

Results – Diagnostic Metrics p.10 2–3 Added NPV, chi-square results, CIs

Results – Risk Stratification p.11 1 Added comprehensive patient flow table

Discussion p.13–15 All Removed superiority claims; added bias discussion

Tables 1–2 p.17–18 Legends Reworded scalability claims; added CIs

Figures 1–3 p.19–21 Legends Clarified metrics, added percentages

# Peer review: Decision Letter 1

**DOI:** 10.1371/journal.pone.0357975.r003

**Authors:** Elingarami Sauli

28 Apr 2026

Dear Dr. Malherbe,

Thank you for submitting your manuscript to PLOS ONE. After careful consideration, we feel that it has merit but does not fully meet PLOS ONE’s publication criteria as it currently stands. Therefore, we invite you to submit a revised version of the manuscript that addresses the points raised during the review process.

==============================

When responding to reviewers' comments, the authors should harmonize/merge the background and introduction sections. The authors should also improve overall reporting of obtained results and their respective discussion, including also highlighting all the limitations of their study in the conclusion part.

==============================

Please submit your revised manuscript by Jun 12 2026 11:59PM. If you will need more time than this to complete your revisions, please reply to this message or contact the journal office at plosone@plos.org. When you're ready to submit your revision, log on to https://www.editorialmanager.com/pone/ and select the 'Submissions Needing Revision' folder to locate your manuscript file.

- A letter that responds to each point raised by the academic editor and reviewer(s). You should upload this letter as a separate file labeled 'Response to Reviewers'.
- A marked-up copy of your manuscript that highlights changes made to the original version. You should upload this as a separate file labeled 'Revised Manuscript with Track Changes'.
- An unmarked version of your revised paper without tracked changes. You should upload this as a separate file labeled 'Manuscript'.

If you would like to make changes to your financial disclosure, please include your updated statement in your cover letter. Guidelines for resubmitting your figure files are available below the reviewer comments at the end of this letter.

If applicable, we recommend that you deposit your laboratory protocols in protocols.io to enhance the reproducibility of your results. Protocols.io assigns your protocol its own identifier (DOI) so that it can be cited independently in the future. For instructions see: https://journals.plos.org/plosone/s/submission-guidelines#loc-laboratory-protocols. Additionally, PLOS ONE offers an option for publishing peer-reviewed Lab Protocol articles, which describe protocols hosted on protocols.io. Read more information on sharing protocols at https://plos.org/protocols?utm_medium=editorial-email&utm_source=authorletters&utm_campaign=protocols.

As the corresponding author, your ORCID iD is verified in the submission system and will appear in the published article. PLOS supports the use of ORCID, and we encourage all coauthors to register for an ORCID iD and use it as well. Please encourage your coauthors to verify their ORCID iD within the submission system before final acceptance, as unverified ORCID iDs will not appear in the published article. *Only*  the individual author can complete the verification step; PLOS staff *cannot*  verify ORCID iDs on behalf of authors.

We look forward to receiving your revised manuscript.

Kind regards,

Elingarami Sauli, PhD

Academic Editor

PLOS One

**Journal Requirements:**

1. If the reviewer comments include a recommendation to cite specific previously published works, please review and evaluate these publications to determine whether they are relevant and should be cited. There is no requirement to cite these works unless the editor has indicated otherwise.

2. Please review your reference list to ensure that it is complete and correct. If you have cited papers that have been retracted, please include the rationale for doing so in the manuscript text, or remove these references and replace them with relevant current references. Any changes to the reference list should be mentioned in the rebuttal letter that accompanies your revised manuscript. If you need to cite a retracted article, indicate the article’s retracted status in the References list and also include a citation and full reference for the retraction notice.

[Note: HTML markup is below. Please do not edit.]

Reviewers' comments:

Reviewer's Responses to Questions

**Comments to the Author**

Reviewer #1: (No Response)

Reviewer #3: (No Response)

**********

2. Is the manuscript technically sound, and do the data support the conclusions?

Reviewer #1: (No Response)

Reviewer #3: Partly

**********

3. Has the statistical analysis been performed appropriately and rigorously?

Reviewer #1: (No Response)

Reviewer #3: No

**********

4. Have the authors made all data underlying the findings in their manuscript fully available?

The PLOS Data policy

Reviewer #1: (No Response)

Reviewer #3: Yes

**********

5. Is the manuscript presented in an intelligible fashion and written in standard English?

Reviewer #1: (No Response)

Reviewer #3: Yes

**********

Reviewer #1: Dear Editor,

The authors have revised their manuscript and dealt with many of my comments on the original submission. I believe the manuscript is now clearer than before. However, there are some comments where the authors have said in their response that they have made a revision, but these revisions aren’t visible in the manuscript (I did at one point wonder if the authors had possibly submitted the wrong version of the revised manuscript). Additionally, some of the revisions made raise further queries and inconsistencies that require clarification. I have the following comments addressed to the authors, which I believe will improve the quality of the manuscript:

1. There is both an Introduction and Background section. Is this intentional? There should be just one (unless the journal has asked you to use this structure)

2. It remains unclear how Breast AI actually works. For example, does it work by looking for tumour cells that aren’t easily visible to a person? It would be helpful if you could please add some explanation of what the tool is doing.

3. The results section says “These findings demonstrate consistent referral performance across diverse healthcare settings when Breast AI™-supported ultrasound assessment was incorporated into routine clinical workflows.” I don’t understand what this means – this is an observational study, so Breast AI played no role in informing care or decision-making, so we don’t expect it to make any difference to referral performance. Or do you mean that the presence of the tool itself did not get in the way of clinical workflows?

4. The results section says that the study time period was from April 2023 to December 2024. What does this mean? Because the dates for each site given in the methods range from July 2023 to April 2025.

5. The authors have said in their response that site-level chi-square results are now reported, where applicable. But I don’t see these anywhere in the manuscript. Could the authors please clarify where exactly they are, or add them in if they have been missed?

6. The ordering of figures is wrong – Figure 2 comes before Figure 1 in the manuscript.

7. The last paragraph in the results section says that “Diagnostic performance of Breast AI™ was evaluated using standard measures of…” But the previous paragraph already presents the diagnostic accuracy results. I am not sure why there is an explanation of what diagnostic accuracy measures were used after already reporting the diagnostic accuracy measure results?

8. There are two Figure 1s – one showing quarters on the x-axis and one showing the risk categories. I believe the latter is the correct one and the former is meant to be removed from the manuscript?

9. Figure 1 is in the discussion but should be in the results – generally, all results should be reported in the results section.

10. There is no citation to Figure 3 anywhere in the text. Line 228-235 suggests figure 2 is the % of cases by pathological site and risk score, but I think this should cite Figure 3, not Figure 2?

11. Some tables also appear in the discussion. It seems the authors have literally just moved entire paragraphs from the results to the discussion (as per my reviewer comments on the previous version) but without correcting for the fact that tables and figures need to be in the results.

12. Thanks for adding Table 3 – this will be helpful for readers. However, the table does not match the results in the manuscript. The table shows 99.4% NPV and this is consistent with what is written in line 260. But where does the NPV of 95.6% in line 244 come from? Table 3 shows 64.7% PPV but line 244 says the PPV was 95.2%? Where did 95.2% come from? Table 3 shows 99.3% sensitivity but line 242 says 93.8%? Where did 93.8% come from? The table shows 69.8% specificity but line 242 says 89.5%? Where did 89.5% come from?

13. The authors have said in their response that 95% confidence intervals have been added to Table 2, but I don’t see these in Table 2, or even in any other table.

14. I would urge the authors to proofread the manuscript. It seems as though they updated some sections based on reviewer comments but did not consistently update the rest of the manuscript. There is also a lot of unnecessary repetition, e.g. the mean age of the cohort, description of the diagnostic accuracy measures assessed, and the fact that Wilson’s binomial 95% CIs were derived is presented in both the methods and results sections.

Reviewer #3: Thank you for the opportunity to review this revised manuscript. Overall, the authors present an important prospective, multi-site “real-world” implementation study of an AI-supported breast ultrasound assessment pathway. The work is clinically relevant—particularly the reported high NPV for low-risk classifications—but key design and verification limitations still constrain the strength of the conclusions and the validity of the reported accuracy estimates.

Here are my comments/recommendations:

1. The study is single-arm: all participants undergo the AI-supported pathway. Without a parallel standard-of-care group (ultrasound without AI) or a clearly defined, site-matched historical cohort, the manuscript cannot quantify benefit attributable to Breast AI™ (diagnostic performance, referral appropriateness, or downstream resource utilization). As a result, any statements implying improved referrals, reduced workload, or superior accuracy are inherently speculative and should be framed strictly as observed performance within this pathway, not as improvement versus usual care.

2. Only moderate- to high-risk patients appear to receive definitive verification via histopathology, whereas low-risk patients are not systematically verified. This differential verification can bias sensitivity/specificity upward and make the reported NPV difficult to validate without follow-up for interval cancers. The manuscript should more prominently explain how differential disease verification distorts diagnostic accuracy estimates and how this affects the interpretation of headline metrics (especially sensitivity and NPV). A key methodological reference here is:

Alonzo TA, Brinton JT, Ringham BM, Glueck DH. Bias in estimating accuracy of a binary screening test with differential disease verification. Statistics in Medicine. 2011;30(15):1852–1864. https://pmc.ncbi.nlm.nih.gov/articles/PMC3115446/

3. The title/discussion imply efficiency gains, but the study does not report efficiency endpoints (radiologist time, workflow throughput, waiting times, costs, biopsy rates vs. the non-AI pathway, downstream imaging, or patient journey metrics). If efficiency is a motivation, it must be explicitly framed as a hypothesis that requires dedicated study designs and outcomes. As written, “efficiency” language risks overstating what the data support.

4. Even acknowledging proprietary constraints, the manuscript should clarify what outputs the system provides (black-box risk score vs interpretable features), how the risk score is presented to clinicians, and how it integrates into workflow (timing, interface, required inputs, failure modes).

5. The five sites likely differ (academic vs community setting, patient demographics, baseline prevalence, radiologist experience, equipment). A more structured site-level description would help readers assess the transferability of results.

6. A prospective diagnostic accuracy study should justify sample size or precision targets for sensitivity/specificity estimates (and/or CI width).

7. The manuscript should explicitly state whether any AI outputs were missing/failed, whether any reference standard results were indeterminate, and how these cases were managed analytically. This matters for real-world feasibility and bias.

8. A STARD-style flow diagram would clarify enrollment, risk stratification counts, referral counts, verification counts, exclusions, losses to follow-up, and final analytic denominators.

9. In a prospective observational study, including a registry listing (or a clear explanation if not registered) enhances transparency.

10. Delays between ultrasound/AI classification and histopathology can introduce bias (progression, interventions, loss to follow-up). This interval should be stated and ideally summarized as median (IQR).

11. Sensitivity and specificity must be reported with CIs (not only NPV). Precision is essential for interpreting performance and comparing it to the literature.

12. The manuscript emphasizes NPV (clinically important), but PPV is critical for biopsy/referral burden. If PPV is low, “over-referral” may occur even with a strong NPV.

13. The authors dichotomize moderate/high as “test-positive,” but clinicians need category-level breakdowns (e.g., cancer rate per category; sensitivity/specificity at different thresholds). The cut-offs must be explicitly defined and justified (pre-specified vs post-hoc), as post-hoc thresholding risks overfitting and weaker generalizability.

14. If this means “proportion of referred patients with cancer,” say so explicitly and acknowledge that it differs from standard diagnostic accuracy constructs. Consider replacing with standard terms (e.g., cancer detection rate among referred, biopsy yield).

15. Specify the biopsy type(s) and whether pathology review was standardized (single vs. multiple pathologists; interobserver process), as variability can affect the reliability of the “gold standard.”

16. Were clinicians blinded to AI initially? How were disagreements handled? Without this, it’s hard to understand whether AI influenced decision-making or merely documented it.

17. Multi-site implementation requires addressing equipment/software differences, training, QA, and calibration. A relevant implementation perspective reference is:

Kotter E, Ranschaert E. Challenges and solutions for introducing artificial intelligence (AI) in daily clinical practice. European Radiology. 2020;31(5):2959–2961. https://pmc.ncbi.nlm.nih.gov/articles/PMC7755626/

18. If “efficiency” remains in the narrative, acknowledge that adoption costs, integration overhead, and downstream savings require dedicated economic evaluation.

19. Consider reporting, or at least discussing, plans for stratification by breast density, age, lesion characteristics (size/morphology/location), and radiologist experience, as these factors plausibly affect ultrasound/AI performance.

20. Without follow-up (e.g., 2–3 years) for low-risk patients, interval cancers cannot be captured, and NPV remains uncertain in practice. Even if not available now, this should be clearly stated as a major limitation and a future work priority.

21. The diagnostic metrics table should include full 2×2 counts and CIs, add TP/TN/FP/FN counts, and provide CIs for sensitivity, specificity, PPV, and NPV; clearly state how the categories were dichotomized.

22. Figures should display uncertainty and improve labeling, Add confidence intervals/error bars, clear y-axis metric labels, and legends that fully explain symbols/colors.

23. Replace broad phrases like “consistent risk stratification” with specific statements (e.g., “similar cancer rates within risk categories across sites” if supported). Also, avoid “impact/efficiency/improvement” language unless it is directly measured.

Best of luck

**********

what does this mean?). If published, this will include your full peer review and any attached files.

If you choose “no”, your identity will remain anonymous but your review may still be made public.

**Do you want your identity to be public for this peer review?**  For information about this choice, including consent withdrawal, please see our Privacy Policy

Reviewer #1: No

Reviewer #3: No

**********

[NOTE: If reviewer comments were submitted as an attachment file, they will be attached to this email and accessible via the submission site. Please log into your account, locate the manuscript record, and check for the action link "View Attachments". If this link does not appear, there are no attachment files.]

To ensure your figures meet our technical requirements, please review our figure guidelines: https://journals.plos.org/plosone/s/figures

You may also use PLOS’s free figure tool, NAAS, to help you prepare publication quality figures: https://journals.plos.org/plosone/s/figures#loc-tools-for-figure-preparation.

NAAS will assess whether your figures meet our technical requirements by comparing each figure against our figure specifications.

# Author comment: Author response to Decision Letter 2

**DOI:** 10.1371/journal.pone.0357975.r004

26 May 2026

Response to Reviewers

Manuscript Title: Transforming Breast Cancer Diagnosis: A Multi-Site Study on the Impact of Breast AI™ in Clinical Assessment, Referral Accuracy, and Healthcare Efficiency

The authors sincerely thank the reviewers and editor for the detailed and constructive feedback. All comments were carefully considered and incorporated to improve the scientific clarity, methodological transparency, and overall academic quality of the manuscript.

Reviewer 1 Comment Author Response Manuscript Revision

1. There is both an Introduction and Background section. There should be just one. We agree with the reviewer. The separate “Background” section has been merged into the Introduction to improve manuscript structure and readability in accordance with standard PLOS ONE formatting conventions. The manuscript now contains a single integrated Introduction section incorporating epidemiology, LMIC context, AI rationale, and study objectives. Previous “Background” heading removed. Pages 3–5, Paragraphs 1–7.

2. It remains unclear how Breast AI actually works. We thank the reviewer for identifying this lack of technical clarity. Additional detail has now been added describing the Breast AI™ workflow, including ultrasound image acquisition, AI-derived probabilistic risk stratification, real-time software integration, clinician-facing outputs, and the adjunctive role of the system in clinical assessment. We also clarified that the system does not directly identify tumour cells, but instead analyses ultrasound-derived imaging features associated with malignancy probability. Expanded methodological description added to the Methods section under “Breast AI™ platform and workflow integration.” Pages 6–7, Paragraphs 2–4.

3. The statement regarding “consistent referral performance” was unclear. We agree and have revised the wording to avoid implying causal improvement in referrals. The revised wording clarifies that the study evaluated observed referral patterns within an AI-supported workflow rather than demonstrating superiority over standard care. Revised Results wording now states: “Referral patterns remained stable across heterogeneous healthcare settings within the observational AI-supported assessment pathway.” Pages 8–9, Paragraphs 1–2.

4. Study dates were inconsistent. We thank the reviewer for identifying this inconsistency. The study period has been corrected and harmonised throughout the manuscript. The overall study period was 1 April 2023 to 30 April 2025, with site-specific commencement dates varying according to ethics approvals and implementation timelines. All inconsistent date references corrected throughout Abstract, Methods, and Results sections. Pages 1, 5, and 9.

5. Site-level chi-square results were not visible. We appreciate this observation. Site-level chi-square analysis results have now been explicitly added to the Results section, including the corresponding p-values. Chi-square outcomes added to Results section and Table 2 legend. Page 9, Paragraph 3.

6. Figure ordering was incorrect. We agree and have corrected the numbering and sequential placement of all figures. Figures reordered throughout manuscript. Figure citations corrected in text.

7. Diagnostic accuracy explanation appeared after reporting results. We agree. The explanatory text regarding sensitivity, specificity, PPV, NPV, dichotomisation, and Wilson confidence intervals has been moved entirely into the Statistical Analysis subsection of the Methods. Redundant methodological explanation removed from Results section and consolidated under Statistical Analysis. Pages 7–8.

8. There were two Figure 1s. We thank the reviewer for identifying this formatting error. The duplicate figure has been removed and all figure numbering has been corrected. Duplicate Figure 1 removed. Remaining figures renumbered sequentially.

9. Figure 1 appeared in the Discussion instead of the Results section. We agree and have relocated all tables and figures to the Results section in accordance with standard reporting conventions. Figures and tables relocated to Results section. Discussion section now contains interpretative commentary only.

10. Figure 3 was not cited correctly. We thank the reviewer for identifying this citation error. Figure citations have now been corrected and aligned with the corresponding text descriptions. Figure 3 is now correctly cited in the pathological subtype risk stratification paragraph. Page 10, Paragraph 2.

11. Some tables appeared in the Discussion section. We agree. All data presentation tables have now been consolidated within the Results section. The Discussion has been revised to avoid repetition of numerical results. Tables 1–4 relocated and discussion rewritten for interpretation-focused narrative. Pages 8–13.

12. Diagnostic accuracy metrics were inconsistent between text and Table 3. We sincerely thank the reviewer for identifying these inconsistencies. All diagnostic performance metrics were recalculated and cross-checked against the underlying 2×2 contingency data. The previously reported sensitivity, specificity, PPV, and NPV values contained transcription inconsistencies between narrative text and tabulated data. These have now been fully corrected and harmonised throughout the manuscript. Corrected diagnostic accuracy metrics are now consistently reported in Abstract, Results, Discussion, and Table 3. Revised values include sensitivity 99.3%, specificity 69.8%, PPV 64.7%, and NPV 99.4%, each with corresponding 95% confidence intervals. Pages 1, 10–12.

13. Confidence intervals were missing from Table 2. We thank the reviewer for identifying this omission. Confidence intervals have now been added to all diagnostic accuracy metrics in the revised tables. 95% confidence intervals added to diagnostic metrics tables and corresponding Results text. Tables 2 and 3 revised.

14. Manuscript required proofreading and removal of repetition. We agree and have undertaken comprehensive language editing and structural revision throughout the manuscript. Repetitive methodological descriptions and duplicated demographic information were removed, while grammar, syntax, and scientific tone were substantially refined to align with PLOS ONE academic standards. Entire manuscript professionally revised for language consistency, conciseness, and scientific clarity. Repetition removed across Methods, Results, and Discussion sections.

Reviewer 3 Comment Author Response Manuscript Revision

1. The study cannot quantify benefit attributable to Breast AI™ without a comparator group. We agree with the reviewer and have substantially moderated all causal or comparative language throughout the manuscript. The revised manuscript now consistently frames the study as an observational implementation study evaluating performance within an AI-supported workflow rather than demonstrating superiority over standard care. Title, Abstract, Results, Discussion, and Conclusion revised to remove unsupported claims regarding “improvement,” “impact,” or superiority. Comparative statements now framed cautiously.

2. Differential verification bias limits interpretation of sensitivity and NPV. We thank the reviewer for this important methodological observation. A dedicated limitations paragraph has now been added discussing verification bias arising from selective histopathological confirmation among moderate- and high-risk groups. We also added discussion regarding the uncertainty of NPV without long-term interval cancer follow-up. The recommended Alonzo et al. reference has been incorporated. Expanded limitations section added to Discussion and Conclusion. Alonzo et al. cited in Discussion. Pages 14–15.

3. Efficiency claims were overstated. We agree and have revised all language referring to workflow efficiency, workload reduction, or operational improvement. Such statements are now explicitly framed as exploratory observations rather than measured outcomes. “Efficiency” wording revised throughout manuscript. Objective statements and conclusions moderated accordingly.

4. Clarify AI outputs and workflow integration. Additional technical detail regarding AI-generated outputs, workflow timing, image acquisition, clinician interface, and adjunctive decision-support integration has now been added. Expanded Methods subsection: “Breast AI™ workflow integration and output generation.” Pages 6–7.

5. More structured site-level descriptions were needed. We agree and have expanded site descriptions to include healthcare setting type, referral environment, patient population context, and resource variability. Site descriptions revised in Methods section. Page 6.

6. Sample size justification was absent. We thank the reviewer for this observation. A sample size justification based on feasibility and precision estimation for diagnostic accuracy outcomes has now been added. Statistical Analysis section updated with sample size rationale. Page 7.

7. Missing AI outputs and indeterminate results should be addressed. We agree and have added explicit reporting regarding missing AI outputs, technical failures, and incomplete histopathology results. Methods and Results sections updated to state that no AI output failures occurred during final analysis and incomplete datasets were excluded prior to analysis.

8. A STARD-style flow diagram was recommended. We appreciate this recommendation and have added a STARD-style participant flow diagram summarising enrolment, exclusions, referrals, histopathological verification, and final analytic cohorts. New Figure 1 added to Results section.

9. Registry listing or explanation should be included. We thank the reviewer for this recommendation. The manuscript now clarifies that the study was conducted as a prospective observational implementation study and was not prospectively registered because it did not involve an interventional therapeutic trial. Registry clarification added to Ethics and Study Design subsection.

10. Time interval between imaging and pathology should be reported. We agree and have now included the median interval between Breast AI™ assessment and histopathological confirmation where available. Median diagnostic interval and IQR added to Results section.

11. Sensitivity and specificity require confidence intervals. We agree and have added 95% confidence intervals for sensitivity, specificity, PPV, and NPV throughout all tables and narrative sections. Diagnostic metrics tables revised accordingly.

12. PPV should be discussed more carefully. We agree and have added discussion regarding the relationship between PPV and referral burden, including the trade-off between maintaining high sensitivity and potential over-referral. Discussion expanded to include implications of PPV and referral burden. Page 14.

13. Dichotomisation thresholds require clarification and justification. We thank the reviewer for identifying this issue. Thresholds are now explicitly defined, and the manuscript clarifies that thresholds were predefined according to the operational Breast AI™ risk stratification framework rather than derived post hoc from the study cohort. Threshold definitions and rationale added to Statistical Analysis section. Page 7.

14. Referral accuracy terminology required clarification. We agree and have revised terminology throughout the manuscript. “Referral accuracy” is now described more precisely as the proportion of referred patients subsequently diagnosed with malignancy. Terminology revised in Results and Discussion.

15. Pathology methods required clarification. Additional details regarding biopsy techniques and pathology review processes have now been added. Histopathology methods subsection added. Page 7.

16. Clinician blinding and disagreement handling required clarification. We thank the reviewer for this important point. The revised manuscript clarifies that clinicians retained full decision-making authority and that Breast AI™ outputs were used adjunctively without overriding clinician judgement. Clarified in Study Design and Workflow sections. Pages 5–6.

17. Multi-site implementation factors should be discussed. We agree and have expanded the Discussion section to address implementation considerations including equipment standardisation, operator training, quality assurance, and calibration. The Kotter and Ranschaert reference has been added. Discussion expanded with implementation considerations. Page 15.

18. Economic implications should be framed cautiously. We agree and have revised all cost and efficiency-related statements to emphasise that dedicated health-economic evaluation remains necessary. Conclusion and Discussion revised accordingly.

19. Stratified analyses should be discussed. We thank the reviewer for this recommendation. Future work plans now include subgroup analyses by breast density, lesion morphology, age, and operator experience. Added to Discussion section.

20. Lack of long-term follow-up limits certainty regarding NPV. We fully agree. This has now been prominently acknowledged as a major study limitation. Expanded limitations section added to Discussion and Conclusion.

21. Diagnostic metrics table should include 2×2 counts and CIs. We thank the reviewer for this recommendation. A full 2×2 diagnostic contingency table has now been incorporated, including TP, TN, FP, and FN counts with corresponding confidence intervals. Revised diagnostic performance table added.

22. Figures should display uncertainty and improved labelling. We agree and have revised all figures to improve axis labels, legends, and explanatory detail. Confidence intervals were added where statistically appropriate. Figures revised accordingly.

23. Broad phrases such as “consistent risk stratification” should be replaced with more precise wording. We agree and have revised the manuscript throughout to ensure wording remains observational, precise, and scientifically supported. Terminology revised throughout manuscript.

# Peer review: Decision Letter 2

**DOI:** 10.1371/journal.pone.0357975.r005

**Authors:** Elingarami Sauli

25 Aug 2026

Multi-Site Observational Evaluation of Breast AI™-Supported Ultrasound Risk Stratification in Breast Cancer Assessment Pathways

PONE-D-25-24224R2

Dear Dr. Kathryn,

We’re pleased to inform you that your manuscript has been judged scientifically suitable for publication and will be formally accepted for publication once it meets all outstanding technical requirements.

Within one week, you’ll receive an e-mail detailing the required amendments. When these have been addressed, you’ll receive a formal acceptance letter and your manuscript will be scheduled for publication.

An invoice will be generated when your article is formally accepted. Please note, if your institution has a publishing partnership with PLOS and your article meets the relevant criteria, all or part of your publication costs will be covered. Please make sure your user information is up-to-date by logging into Editorial Manager at Editorial Manager® and clicking the ‘Update My Information' link at the top of the page. For questions related to billing, please contact billing support.

If your institution or institutions have a press office, please notify them about your upcoming paper to help maximize its impact. If they’ll be preparing press materials, please inform our press team as soon as possible -- no later than 48 hours after receiving the formal acceptance. Your manuscript will remain under strict press embargo until 2 pm Eastern Time on the date of publication. For more information, please contact onepress@plos.org.

Kind regards,

Elingarami Sauli, PhD

Academic Editor

PLOS One

Additional Editor Comments (optional):

The authors have made significant and satisfactory revisions to this submission, which can now be accepted for publication.

Reviewers' comments:

Reviewer's Responses to Questions

**Comments to the Author**

Reviewer #1: (No Response)

**********

2. Is the manuscript technically sound, and do the data support the conclusions?

Reviewer #1: (No Response)

**********

3. Has the statistical analysis been performed appropriately and rigorously?

Reviewer #1: (No Response)

**********

4. Have the authors made all data underlying the findings in their manuscript fully available?

The PLOS Data policy

Reviewer #1: (No Response)

**********

5. Is the manuscript presented in an intelligible fashion and written in standard English?

Reviewer #1: (No Response)

**********

Reviewer #1: Dear Co-authors,

Thanks for revising the manuscript and dealing with many of my comments on revision 1. I believe the manuscript is now clearer than before. However, some of the revisions raise further queries and inconsistencies that require clarification:

1. Response to review 1 comment 5 says that site-level chi-square results have been added to the Results section and Table 2 legend. Thanks for adding these to the Results section, but I do not see these in the Table 2 legend.

2. You have added in the Methods section that a sample size/power calculation was not performed because the study was designed as an observational implementation evaluation rather than a comparative interventional trial. A study doesn’t need to be a comparative interventional trial in order to do a calculation. We usually do sample size calculations for observational studies too, so I don’t understand this statement.

3. The last paragraph of the Methods section starts with “Prior validation studies reported a diagnostic accuracy of 97.6% for the Breast AI™ system under development and testing conditions.” Could you please provide a reference for this. Also how is "accuracy" defined here?

4. In the Results section, paragraph two, it says that “Site-level referral-associated malignancy rates ranged from 95.6% at the tertiary referral hospital (Site 5)…” but Table 2 shows 95.7%, not 95.6%. Which is correct?

5. In the Results section, paragraph two, it says that “Site-level referral-associated malignancy rates ranged from 95.6% at the tertiary referral hospital (Site 5) to 100% at both the private diagnostic clinic (Site 2) and the community-based clinic setting (Site 1) (Table 1).” I believe this should refer to Table 2, not Table 1?

6. In Table 1, there is an asterisk in column 2 row 3. What does this asterisk refer to? There is no corresponding statement/footnote linked to this asterisk.

7. In Table 4, the NPV is missing.

**********

what does this mean?). If published, this will include your full peer review and any attached files.

If you choose “no”, your identity will remain anonymous but your review may still be made public.

**Do you want your identity to be public for this peer review?**  For information about this choice, including consent withdrawal, please see our Privacy Policy

Reviewer #1: No

**********

# Editor report: Acceptance letter

**DOI:** 10.1371/journal.pone.0357975.r006

**Authors:** Elingarami Sauli

PONE-D-25-24224R2

PLOS One

Dear Dr. Malherbe,

I'm pleased to inform you that your manuscript has been deemed suitable for publication in PLOS One. Congratulations! Your manuscript is now being handed over to our production team.

At this stage, our production department will prepare your paper for publication. This includes ensuring the following:

* All references, tables, and figures are properly cited

* All relevant supporting information is included in the manuscript submission,

* There are no issues that prevent the paper from being properly typeset

You will receive further instructions from the production team, including instructions on how to review your proof when it is ready. Please keep in mind that we are working through a large volume of accepted articles, so please give us a few days to review your paper and let you know the next and final steps.

Lastly, if your institution or institutions have a press office, please let them know about your upcoming paper now to help maximize its impact. If they'll be preparing press materials, please inform our press team within the next 48 hours. Your manuscript will remain under strict press embargo until 2 pm Eastern Time on the date of publication. For more information, please contact onepress@plos.org.

You will receive an invoice from PLOS for your publication fee after your manuscript has reached the completed accept phase. If you receive an email requesting payment before acceptance or for any other service, this may be a phishing scheme. Learn how to identify phishing emails and protect your accounts at https://explore.plos.org/phishing.

If we can help with anything else, please email us at customercare@plos.org.

Thank you for submitting your work to PLOS One and supporting open access.

Kind regards,

PLOS One Editorial Office Staff

on behalf of

Dr. Elingarami Sauli

Academic Editor

PLOS One
